# Primary renal primitive neuroectodermal tumour causing Budd–Chiari syndrome: a rare case report

**DOI:** 10.1259/bjrcr.20150184

**Published:** 2016-11-02

**Authors:** Biswajit Mishra, SH Chandrashekhara, Smita Manchanda, Sudheer Arava

**Affiliations:** ^1^Department of Radiodiagnosis, All India Institute of Medical Sciences, New Delhi, India; ^2^Department of Pathology, All India Institute of Medical Sciences, New Delhi, India

## Abstract

Primary Ewing’s sarcoma/primitive neuroectodermal tumour (ES/PNET) of the kidney is a rare neoplasm characterized by an aggressive clinical course. Most often, ES/PNET arises in the chest wall and paraspinal locations, and less commonly in the limbs or the genitourinary tract. We present a rare case of renal ES/PNET presenting as Budd–Chiari syndrome.

## Summary

Ewing’s sarcoma/primitive neuroectodermal tumour (ES/PNET) of the kidneys are extremely rare; to date, only 50 cases have been reported in the English literature. They tend to affect young adults, with a slight male predominance.^1^ Previously, ES and PNET were considered separate entities but recent pathological/molecular studies have established that they belong to same tumour family and exhibit similar behaviour.^2^ ES/PNET are most commonly located in the chest wall and paraspinal region; less common locations described are the limbs and the genitourinary tract.^3^ Here, we report a rare case of renal ES/PNET presenting as Budd–Chiari syndrome.

## Case presentation

A 16-year-old male patient came to our hospital with a history of left flank pain for 3 months, two to three episodes of gross haematuria and abdominal distension for the preceding 2 weeks. Pain was described as a dull ache, non-radiating in nature, and not associated with fever or burning during micturition. On examination, the patient was of average built, having no icterus or pallor, and had distension of the abdomen with shifting dullness. Serum glutamic oxaloacetic transaminase (49 U l^−1^), serum glutamic pyruvic transaminase (37 U l^−1^) and bilirubin (0.6 mg%) levels were normal, and prothrombin time was prolonged (20 s). Blood haemogram and renal function tests were within normal limits.

**Figure 1.**
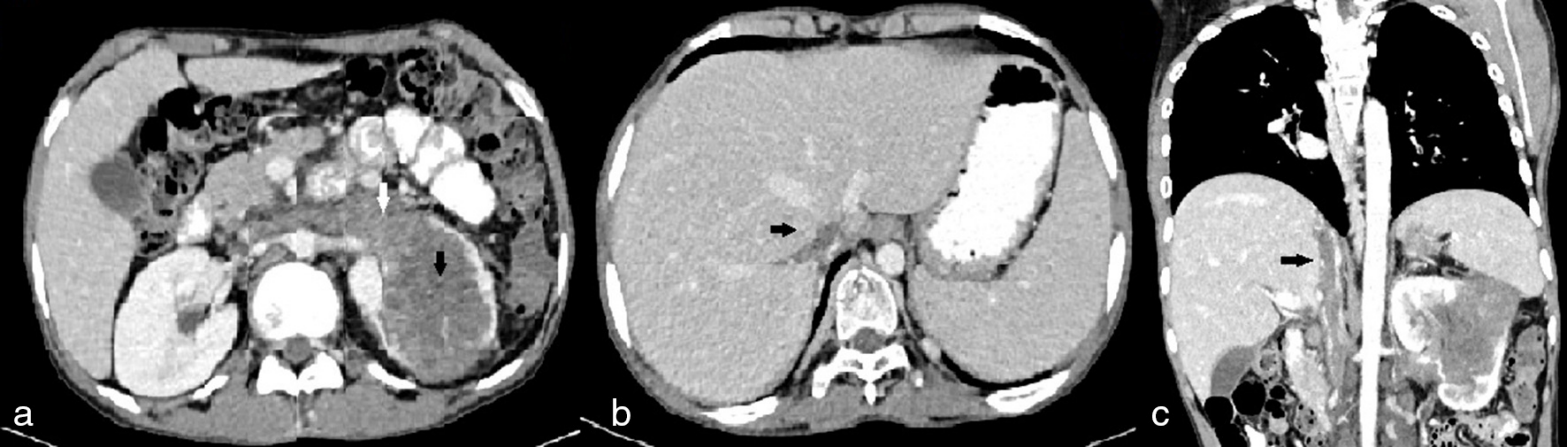
Contrast-enhanced CT scan of a 16-year-old male patient with renal Ewing’s sarcoma/primitive neuroectodermal tumour. (a) Axial section showing a large heterogeneous soft tissue mass (black arrow) arising from the left kidney having areas of necrosis within and extending into the renal vein (white arrow). (b) Axial section reveals the tumour thrombus extending into the inferior vena cava and right hepatic vein (black arrow). (c) Coronal reconstruction image demonstrating the left renal mass with tumour thrombus extending along the inferior vena cava (black arrow).

**Figure 2.**
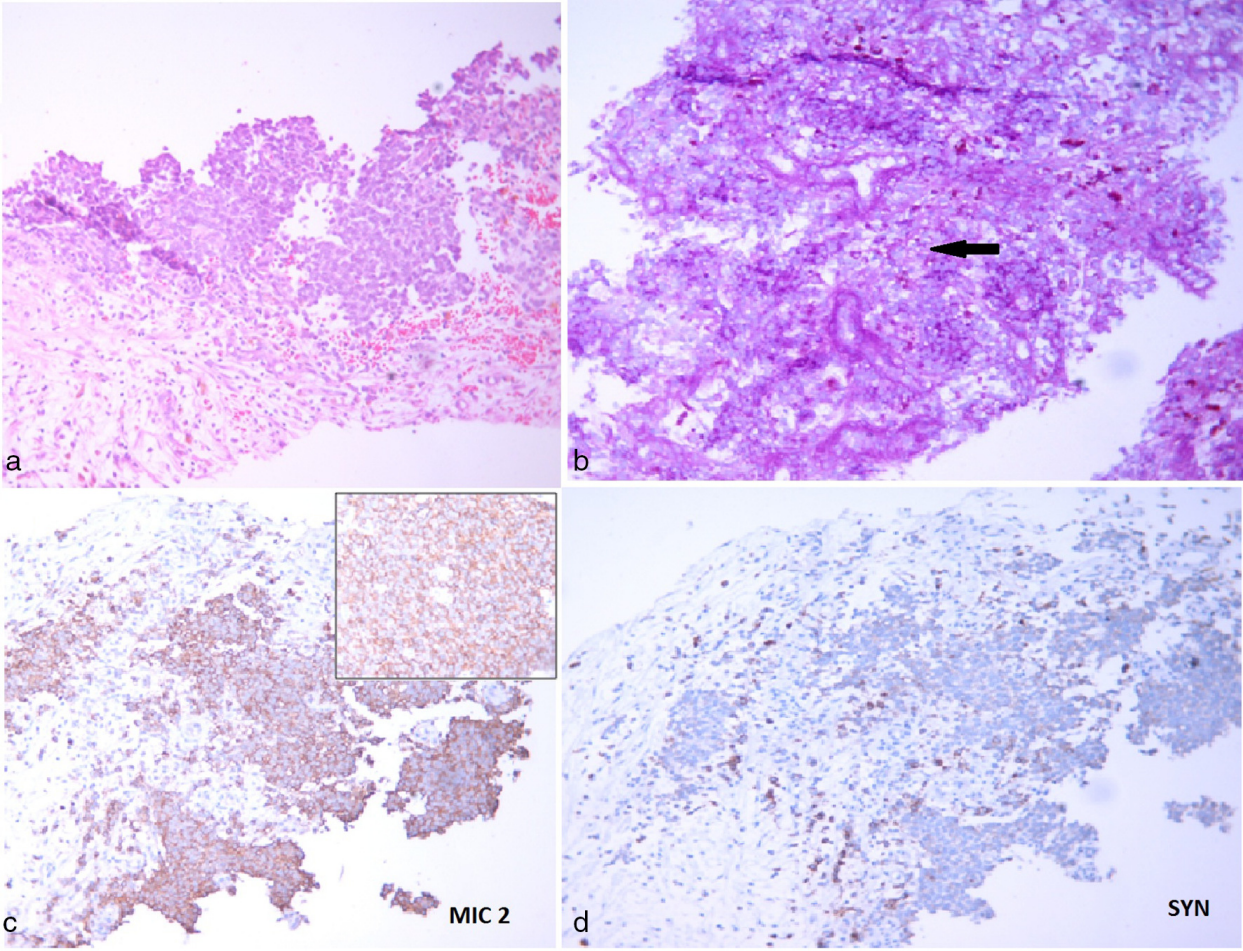
Histopathology of a 16-year-old male patient with renal Ewing’s sarcoma/primitive neuroectodermal tumour. (a) Haematoxylin and eosin stain showing malignant small, round cell tumour morphology. Renal parenchyma is not identified. (b) Some of the tumour cells show periodic acid–Schiff-positive diastase-resistant cytoplasmic granules (arrow). On immunohistochemistry, these cells show (c) strong membranous immunopositivity for cluster of differentiation 99 (box shows a high power view of the same image) and (d) focal immunopositivity for synaptophysin.

Transabdominal ultrasound imaging revealed a hypoechoic mass in the left kidney in the interpolar region, with extension of the tumour to the left renal vein and inferior vena cava (IVC). Contrast-enhanced CT scan of the abdomen showed a large lobulated heterogeneously enhancing mass of size 10.5 × 7 × 5 cm arising from the left kidney with infiltration of adjacent pararenal fat and the retroperitoneum (Figure 1a). Enhancing tumour thrombus was seen extending into the IVC through the left renal vein in continuity with the primary renal mass. Superiorly, the thrombus extended into the intrahepatic portion of the IVC and the right hepatic vein, causing the Budd–Chiari syndrome (Figure 1b,c). There was homogeneous enhancement of the liver parenchyma with normal opacification of the left and middle hepatic veins, and the portal vein. There was mild ascites. An imaging diagnosis of Stage III (T3bN0M0) renal cell neoplasm was made. Biopsy of the mass demonstrated monomorphic small, round cells arranged in a sheet-like pattern with round nuclei, scanty eosinophilic cytoplasm and indistinct cell outline (Figure 2a,b). Immunohistochemistry revealed positivity for cluster of differentiation 99 (MK2), vimentin and synaptophysin (Figure 2c,d). Based on these findings, a diagnosis of renal ES/PNET was established.

## Treatment

The patient is undergoing chemotherapy with vincristine, doxorubicin and cyclophosphamide alternating with ifosfamide and etoposide, and is planned for surgery (radical nephrectomy with caval thrombectomy).

## Discussion

Primary ES/PNET tends to occur in young adults, with a male predominance. Patients usually remain asymptomatic, with the tumour reaching a large size. Usual presenting symptoms are flank pain (85%), palpable mass (60%) and haematuria (37%).^4^ Some rare presentations include Budd–Chiari syndrome,^5,6^ pulmonary thromboembolism^3^ and renal failure.^7^ Renal PNET presenting as Budd–Chiari syndrome is extremely rare. To the best of our knowledge, only two cases have been described to date in the literature. The first case was that of a 17-year-old female with a 14 cm renal mass with tumour extension to the renal vein and IVC, for which she had undergone nephrectomy but the caval thrombus was left behind. She died of Budd–Chiari syndrome and liver failure 5 months post surgery.^5^ The second case was also of a 17-year-old female who had right renal PNET with IVC and hepatic venous thrombus extension. She underwent nephrectomy and caval thrombectomy under hypothermic circulatory arrest after three cycles of neoadjuvant chemotherapy. There was no recurrence in the 7-month follow-up period.^6^

Imaging characteristics of PNET are non-specific and similar to other renal neoplasms, including renal cell carcinoma, Wilm’s tumour, lymphoma, renal sarcoma and metastatic carcinoma. On ultrasound, renal PNET may appear hypo- or hyperechoic to adjacent renal parenchyma and usually show markedly increased vascularity on colour Doppler images. CT studies often show a large heterogeneous mass having areas of necrosis, calcification and haemorrhage. In comparison to other renal neoplasms, areas of haemorrhage and necrosis in ES/PNET are more commonly located at the periphery of the mass.^2^ On MRI, the tumour appears hypointense on *T*_1_ and hyperintense on *T*_2_ weighted images, and shows restricted diffusion. On gadolinium administration, it shows heterogeneous enhancement, depending on the amount of necrosis. In fact, ES/PNET has a higher rate of renal vein thrombosis and distant metastasis than conventional renal cell carcinoma at the time of initial diagnosis.^2^ Imaging differentiation of a benign thrombus from a tumour thrombus is necessary because of dismal surgical and prognostic outcomes of the latter. Tumour thrombus usually causes expansion of the lumen, shows a thread and streak pattern of enhancement in the arterial phase and always shows continuity with the primary thrombus. Internal vascularity on Doppler study and diffusion restriction are also found in the tumour thrombus but presence of IVC infiltration is the most specific sign. Usual sites of metastasis include liver, lungs and bone. Imaging modalities such as ultrasound, CT scan and MRI not only help in diagnosis but also in metastatic evaluation and monitoring treatment response.

In view of the overlapping imaging features of PNET and other renal neoplasms, diagnosis of these tumours is often made by pathological examination. In line with radiology, histopathological features of renal PNET often overlap with other round cell neoplasms of the kidneys such as lymphoma, Wilm’s tumour and neuroblastoma. Immunohistochemistry and molecular studies therefore are very useful in differentiating PNET from these entities. PNET shows positivity for cluster of differentiation 99, friend leukemia integration 1 transcription factor, vimentin and synaptophysin, and is negative for pankeratin, desmin, Wilm’s tumour 1, glial fibrillary acidic protein and paired box gene 2 protein. Molecular studies typically reveal a characteristic ES/friend leukemia integration 1 transcription factor fusion product that results from t(11;22) (q24;q12) translocation and is identified in 90% of cases.^8^

Treatment of renal PNET includes surgery, chemotherapy and radiation therapy. Surgical options include nephrectomy with caval thrombectomy in case of involvement of renal vein/IVC.^2^ Multidrug chemotherapy is usually given as adjuvant therapy after histopathological examination of the resected tissue. There is no absolute protocol of treatment of renal PNET owing to the relative rarity of the tumour. So the treatment options are the same as PNET in other sites. The most commonly used drug combinations include vincristine, actinomycin D, cyclophosphamide alternating with ifosfamide and etoposide.^9^ In spite of aggressive treatment options, the 5-year disease-free survival rate of renal PNET is 45–55%.^8^

## Learning points

1. Renal PNET, though a rare neoplasm, should be considered in the differentials of renal masses, especially in young adults with tumour extension along the IVC.
2. Although immunohistochemistry plays an important role in the diagnosis of PNET, imaging studies have a crucial role in predicting disease extent of the primary tumour and tumour thrombus.
3. No absolute treatment protocol is available. Surgical options include nephrectomy with caval thrombectomy in case of renal vein/IVC involvement, usually followed by adjuvant chemotherapy.

## Consent

Informed consent from the patient, guardian or next of kin for the case to be published (including images, case history and data) could not be obtained. Exhaustive efforts were made to contact the patient, guardian or next of kin over a period of 1 year but proved unsuccessful. Patient data has been anonymized to protect patient identity.
